# SARS-CoV-2 seroprevalence and associated factors, based on HIV serostatus, in young people in Sofala province, Mozambique

**DOI:** 10.1186/s12879-023-08808-6

**Published:** 2023-11-17

**Authors:** Roberto Benoni, Virginia Casigliani, Annachiara Zin, Dara Giannini, Niccolò Ronzoni, Costanza Di Chiara, Kajal Chhaganlal, Daniele Donà, Ada Merolle, Helga Guambe Dos Anjos, Fernando Chenene, Francesca Tognon, Giovanni Putoto, Carlo Giaquinto

**Affiliations:** 1 Division of Pediatric Infectious Diseases, Department of Women’s and Children’s Health, University of Padua, Padua, Italy; 2 Doctors with Africa CUAMM Mozambique, Beira, Mozambique; 3 Department of Diagnostics and Public Health, University of Verona, Strada Le Grazie, Verona, 8 – 37134 Italy; 4 Department of Translational Research and of New Surgical and Medical Technologies, University of Pisa, Pisa, Italy; 5 Department of Infectious-Tropical Diseases and Microbiology, IRCCS Sacro Cuore Don Calabria Hospital, Verona, Italy; 6 Faculdade de Ciências de Saúde, Universidade Católica de Moçambique, Beira, Mozambique; 7 UNICEF Mozambique, Maputo, Mozambique; 8 Operational Research Unit, Doctors with Africa CUAMM, Padua, Italy

**Keywords:** SARS-CoV-2, Seroprevalence, COVID-19, HIV, Mozambique, YPLHIV

## Abstract

**Introduction:**

In Sofala province (Mozambique), young people living with HIV (YPLHIV) are estimated at 7% among people aged 15–24 years. Even though the COVID-19 pandemic threatened HIV health services, data on the impact of COVID-19 on YPLHIV people are lacking. This study aimed at exploring the seroprevalence of SARS-CoV-2 and associated factors among young people based on their HIV status.

**Methods:**

A cross-sectional study was conducted, including people aged 18–24 attending a visit at one of the adolescent-friendly health services in Sofala province between October and November 2022. People vaccinated against SARS-COV-2 or YPLHIV with WHO stage III-IV were excluded. A SARS-CoV-2 antibodies qualitative test and a questionnaire investigating socio-demographic and clinical characteristics were proposed. SARS-CoV-2 seroprevalence was calculated with Clopper-Pearson method. The odds ratio (OR) of a positive SARS-CoV-2 antibodies test was estimated through multivariable binomial logistic regression.

**Results:**

In total, 540 young people including 65.8% women and 16.7% YPLHIV participated in the survey.. The mean age was 20.2 years (SD 2.0). Almost all the sample (96.1%) reported adopting at least one preventive measure for COVID-19. The weighted seroprevalence of SARS-CoV-2 in the whole sample was 46.8% (95%CI 42.6–51.2) and 35.9% (95%CI 25.3–47.5) in YPLHIV. The adjusted OR of testing positive at the SARS-CoV-2 antibodies test was higher in students compared to workers (aOR:2.02[0.95CI 1.01–4.21]) and in those with symptoms (aOR:1.52[0.95CI 1.01–2.30]). There were no differences based on HIV status(aOR:0.663[95%CI 0.406–1.069]). Overall, COVID-19 symptoms were reported by 68 (28.2%) people with a positive serological SARS-CoV-2 test and by 7 (21.7%) YPLHIV (*p* = 0.527). No one required hospitalization.

**Conclusions:**

SARS-CoV-2 seroprevalence was 46.8% without differences in risk of infection or clinical presentation based on HIV status. This result may be influenced by the exclusion of YPLHIV with advanced disease. The higher risk among students suggests the schools’ role in spreading the virus. It’s important to continue monitoring the impact of COVID-19 on YPLHIV to better understand its effect on screening and adherence to treatment.

**Supplementary Information:**

The online version contains supplementary material available at 10.1186/s12879-023-08808-6.

## Introduction

The Human Immunodeficiency Virus (HIV) represents a global public health emergency, despite the first clinical case being identified more than 40 years ago.

In 2021, 38.4 million people were living with HIV (PLHIV) worldwide, of whom 9.7 million were not on treatment due to a lack of knowledge of their serological status or difficulties in accessing therapy. In the same year, there were an average of 4,000 new cases per day, 60% in sub-Saharan Africa with 30% concentrated in the 15–24 age group [1, 2].

In 2021, Mozambique registered 94,000 new cases of HIV, making it the second country in sub-Saharan Africa with the highest number of infections. In the same year, it is estimated that there were 1,976,250 PLHIV in the country [2, 3]. In the province of Sofala, in Beira, HIV prevalence is estimated at 7% among adolescents between 15 and 24 years of age [4].

The COVID-19 pandemic hit the African continent, albeit more lightly than other continents, regarding disease severity and mortality. However, the spread of infection is not easily assessed due to the difficulties of surveillance and contact tracing systems and the limited capacity of the necessary laboratory facilities. For this reason, prevalence studies based on the detection of antibodies against SARS-CoV-2 are essential to understand the real spread of the virus in the African continent and, thus, guide public health policies. According to the last available systematic review, the estimated pooled prevalence in Africa, linked to infection or vaccination, reached 65% in September 2021, with a prevalence of 56.1% in southern Africa; however, most studies were only carried out in South Africa [5, 6].

In Mozambique, confirmed cases of SARS-CoV-2 infection were approximately 233,000 on 27/02/2023, with 2,242 deaths [7]. However, the country has had limitations in testing capacity during all the pandemic period: serological studies are therefore essential to better understand which was the real extent of the spread of the virus [8].

The COVID-19 pandemic had a double impact on PLHIV: on the one hand, the risk of contracting SARS-CoV-2 infection and developing a serious illness, on the other hand, the reduction of services with consequent difficulty in access to antiretroviral treatment [9]. During the first year of the pandemic, the HIV testing and antiretroviral therapy (ART) initiation showed a significant decrease, especially among children and adolescents, while the provision of the treatment and the retention in care of PLHIV seemed to be less affected by the pandemic [10]. It may be explained by the multiple interventions adopted, such as the multi-month dispensing of ART [11].

Currently, in low- and middle-income countries (LMIC), and particularly in sub-Saharan Africa, there are not enough studies on the epidemiology of COVID-19 in children and young people living with HIV, nor are there enough data on risk factors and disease outcomes in this population. Therefore, it became a priority to carry out a seroepidemiological study to estimate target group immunity, an essential indicator of the spread of infection in the community, to understand the challenges in accessing and retaining treatment associated with COVID-19.

The aim of the study was to estimate the seroprevalence of antibodies against SARS-CoV-2 at the end of 2022 and to explore its associated factors in young people living with HIV (YPLHIV) who attended the healthcare facilities in the province of Sofala in Mozambique.

## Methods

### Study design, population and intervention

A cross-sectional study was conducted to estimate the prevalence of SARS-COV-2 antibodies in young people accessing healthcare centers in the province of Sofala. We included all young people aged 18 to 24, who attended the adolescent-friendly service within the healthcare centers in Beira City and Nhamatanda (Mozambique) from October to November 2022, regardless of their HIV status. Inclusion criteria to participate in the study were the following: signing the informed consent, not presenting acute symptoms of SARS-COV-2 at the time of the test, not being vaccinated against SARS-COV-2, not being pregnant, not having been diagnosed in the last 6 months and not presenting an advanced clinical stage of HIV (WHO stage III-IV or CD4 < 200/ < 15%). Each participant underwent a questionnaire performed by a trained healthcare worker and had a rapid serological SARS-CoV-2 test done with a capillary blood test.

### Study setting

All eight SAAJs of the Beira district, where Doctors with Africa CUAMM work, and the SAAJ of Nhamatanda Rural Hospital were selected. The city of Beira and the district of Nhamatanda are in the province of Sofala, in the central area of Mozambique. It has an estimated population of 2,528,442, of which 897,467 (35.5%) are aged between 10 and 24 years [12]. For this age group, the government of Mozambique has a special adolescent-friendly service within the health centers that provides education, prevention, and treatment for adolescents and young people, called Serviços amigos dos adolescentes e jovens (SAAJ).

### Sample size calculation

A sample size of 540 youth was calculated using the following formula:

$${\mathrm{z}}^{2}*\mathrm{P }(1-\mathrm{P})/{\Delta }^{2}$$

where z refers to the confidence level (set at 95%, so z = 1.96), P is the estimated prevalence of individuals testing positive (*P* = 0.40, based on the latest available seroprevalence in Beira and Omicron data from South Africa), and Δ is the desired precision (set at 5%) [9].

Considering a dropout around 10%, the final sample size was projected to be 540 participants, allowing an estimated prevalence of 40% (with a 95% confidence interval of 36–44%) adjusted by the sensitivity (96.7%) and specificity (97%) of the diagnostic test as reported by the manufacturer [13].

### Endpoints

The primary endpoint of the study was the prevalence of SARS-CoV-2 antibodies in young people aged 18–24 years who access SAAJ of Beira and Nhamatanda, performed with a rapid diagnostic test. Secondary endpoints were (I) odds ratio of a positive serological test and (II) the number and type of symptoms of SARS-CoV-2 infection and hospitalizations, stratified by HIV status.

### Data collection

A qualitative serological analysis to identify SARS-CoV-2 serostatus was performed through the IgM/IgG rapid test by lateral flow immunochromatographic method (Panbio™ COVID-19 IgG/IgM Rapid Test Duo, Abbott Laboratories, Orlando, USA).

A questionnaire (Additional file) was used to collect sociodemographic characteristics (age, sex, religion, profession, level of education, house density, defined as the number of persons living in the house divided by the number of house rooms, and area of residence), adhesion to SARS-CoV-2 preventive measures, COVID-19 epidemiological information (e.g., COVID-19 cases in the family), and clinical characteristics (i.e., HIV status, comorbidities, COVID-19 related symptoms in the previous 8 months).

The questionnaire was administered in the official local language (Portuguese) by the medical officer of the SAAJ. Data were collected from October to November 2022 and were entered into the REDcap platform to set up the database for subsequent analysis [14].

### Ethical approval

The research was performed following the ethical standards of the 1964 Declaration of Helsinki and was approved by the Interinstitutional Bioethics Committee for Health (Comité Interinstitucional de Bioética para Saúde - CIBS), Sofala, on the 29th of August 2022 (protocol number 002/CIBIS/2022).

### Statistical analysis

A descriptive statistical analysis was performed. Frequencies and proportions were calculated for categorical variables; means and standard deviation were used for continuous data. Sample distribution was tested via χ2 and Fisher exact test for categorical variables or ANOVA test for continuous variables, as appropriate.

Unweighted and weighted estimates of seroprevalence of SARS-CoV-2 with 95% CI were calculated with Clopper Pearson method as the number of positive test results divided by the total number of tests performed during the survey period. The weighted estimate was obtained with the Rogan-Gladen estimator considering a sensitivity and specificity of 96.7% and 97.0%, respectively, according to data provided by the manufacturer.

The univariate association between individual characteristics and seropositivity was explored using the χ2 test and multivariable associations by binomial logistic regression, including all variables found to be statistically significant in univariate analyses. Missing data were handled through pairwise deletion.

A *p*-value < 0.05 was considered significant. All analyses were performed using the R software (version 4.1.1).

## Results

### Patients’ characteristics

Of the 540 young people included, 355 (65.7%) were females. The mean age was 20.2 years (SD 2.0). YPLHIV were 90/540 (16.7%, females = 57/90, 63.3%), with no differences compared with those without HIV based on sex and age. Socio-demographic characteristics are reported in Table 1.

**Table 1:** Sociodemographic characteristics of the sample by HIV status

	**HIV- (*n* = 450)**	**HIV + (*n* = 90)**	*p*-Value*	**Overall (*n* = 540)**
**Sex**			0.685	
Females	298 (66.2%)	57 (63.3%)		355 (65.7%)
Males	152 (33.8%)	33 (36.7%)		185 (34.3%)
**Age (years)**			0.515	
Mean (sd)	20.2 (1.9)	20.4 (2.1)		20.2 (2.0)
**School**			0.026	
None	8 (1.8%)	0 (0.0%)		8 (1.5%)
Primary	54 (12.0%)	20 (22.2%)		74 (13.7%)
Secondary	329 (73.1%)	64 (71.1%)		393 (72.8%)
University	59 (13.1%)	6 (6.7%)		65 (12.0%)
**Job**			0.002	
Workers	29 (6.4%)	16 (17.8%)		45 (8.3%)
Unemployed/ temporary job	183 (40.7%)	38 (42.2%)		221 (40.9%)
Student	238 (52.9%)	36 (40.0%)		274 (50.7%)
**District**			0.843	
Rural	44 (9.8%)	10 (11.1%)		54 (10.0%)
Suburban	300 (66.7%)	61 (67.8%)		361 (66.9%)
Urban	106 (23.6%)	19 (21.1%)		125 (23.1%)
**Religion**			0.318	
Protestant/Anglican	241 (53.6%)	44 (48.9%)		285 (52.8%)
Catholic	99 (22.0%)	26 (28.9%)		125 (23.1%)
None	76 (16.9%)	14 (15.6%)		90 (16.7%)
Muslim	22 (4.9%)	6 (6.7%)		28 (5.2%)
Zione/Sião	12 (2.7%)	0 (0.0%)		12 (2.2%)
**Housemates**			0.737	
0–3	149 (33.1%)	26 (28.9%)		175 (32.4%)
4–5	151 (33.6%)	32 (35.6%)		183 (33.9%)
> 6	150 (33.3%)	32 (35.6%)		182 (33.7%)
**Rooms**			0.607	
1	81 (18.0%)	21 (23.3%)		102 (18.9%)
2	137 (30.4%)	26 (28.9%)		163 (30.2%)
3	155 (34.4%)	31 (34.4%)		186 (34.4%)
> 3	77 (17.1%)	12 (13.3%)		89 (16.5%)
**Children**			0.205	
None	340 (75.6%)	75 (83.3%)		415 (76.9%)
1	100 (22.2%)	15 (16.7%)		115 (21.3%)
2	10 (2.2%)	0 (0.0%)		10 (1.9%)
**Comorbidities**			0.553	
No	431 (95.8%)	88 (97.8%)		519 (96.1%)
Yes	19 (4.2%)	2 (2.2%)		21 (3.9%)

^*^Fisher’s exact and χ2 test, Student’s t-test

Most patients had a middle school degree (*n* = 393/540, 72.8%). YPLHIV more frequently had a lower level of education than HIV-negative patients (*p* = 0.026, Table 1). YPLHIV were more frequently workers (17.8%) and fewer students (40.0%) than those without HIV (*p* = 0.002, Table 1).

### SARS-CoV-2 prevalence

During the study period, 253/540 (46.9%) people tested positive with the rapid serological test for SARS-CoV-2 antibodies. The weighted seroprevalence of SARS-CoV-2 in the whole sample was 46.8% (95%CI 42.6%-51.2%). The seroprevalence by antibodies type was: 1.1% (95%CI 0.0–3.3) for IgM, 1.9% (95%CI 0.2%-4.2%) for IgM + IgG and 37.3% (95%CI 32.9%-41.8%) for IgG. The adjusted seroprevalence was 35.9% (95%CI 25.3%-47.5%) and 49.1% (95%CI 44.1%-54.1%) in the HIV+ and HIV- groups, respectively.

In the univariate analysis, there was no association between a positive test result and sex, age, house density, district or educational level (Table 2). In the multivariable analysis a positive result at the SARS-CoV-2 antibodies test was associated with both job type and COVID-19 symptoms. The OR was higher in students compared to workers (aOR: 2.02 [95%CI: 1.01–4.21]) and in those with symptoms (aOR: 1.52 [95%CI 1.01–2.30]). There were no statistically significant differences based on HIV status (aOR: 0.663 [95%CI: 0.406–1.069], Table 2).

**Table 2:** Results of the univariable (odds ratio - OR) and multivariable (adjusted odds ratio - aOR) logistic regression models fitted on the results at SARS-CoV-2 serological test as dependent variables and HIV status, presence of COVID-19 symptoms and job as potential determinants

	**OR [0.95CI]**	**aOR**	**0.95 CI**
**Positive SARS-CoV-2 test (yes/no)**			
HIV status (+)	0.603 [0.375–0.956]	0.663	0.406–1.069
COVID-19 symptoms (yes)	1.544 [1.030–2.323]	1.525	1.014–2.303
Job (worker)			
Student	2.572 [1.322–5.274]	2.018	1.014–4.206
Unemployed	2.051 [1.042–4.246]	1.729	0.861–3.633
Sex (M)	0.910 [0.637–1.300]		
Age (years)	0.964 [0.884–1.051]		
School (none)			
Primary	0.780 [0.172–3.531]		
Secondary	0.854 [0.199–3.657		
University	1.241 [0.272–5.664		
District (rural)			
Sub-urban	1.760 [0.977–3.25]		
Urban	1.406 [0.730–2.764]		
House density	0.948 [0.810–1.107]		

### SARS-CoV-2 symptoms

COVID-19 symptoms were reported by 68/241 people (28.2%, NA = 12) with positive results at the SARS-CoV-2 antibodies test, with no differences based on HIV status (*p* = 0.527). Symptoms are shown in Table 3.

**Table 3:** Number and frequency of different types of COVID-19 preventive measures used and COVID-19 symptoms distinguished by HIV status

	**HIV- (*n* = 450)**	**HIV + (*n* = 90)**	*p*–Value*	**Overall (*n* = 540)**
**COVID-19 preventive measures**				
Wearing a mask	430 (95.6%)	86 (95.6%)	0.998	516 (95.6%)
Keeping distance	191 (42.4%)	36 (40.0%)	0.726	227 (42.0%)
Avoiding crowded areas	167 (37.1%)	28 (31.1%)	0.336	195 (36.1%)
Hand wash	374 (83.1%)	77 (85.6%)	0.643	451 (83.5%)
	**HIV- (*n* = 220)**	**HIV + (*n* = 33)**		**Overall (*n* = 253)**
**COVID-19 Symptoms**^**a**^** (N/A = 12)**				
Total with symptoms	61 (29.2%)	7 (21.9%)	0.527	68 (28.2%)
Fever	47 (22.5%)	6 (18.8%)	0.820	53 (22.0%)
Cough	49 (23.4%)	6 (18.8%)	0.821	55 (22.8%)
Sore throat	38 (18.2%)	3 (9.4%)	0.315	41 (17.0%)
Anosmia	14 (6.7%)	1 (3.1%)	0.701	15 (6.2%)
Breathing difficulty	3 (1.4%)	2 (6.3%)	0.128	5 (2.1%)
Hospitalization	0 (0.0%)	0 (0.0%)	0.998	0 (0.0%)

^*^Fisher’s exact and χ2 test

^a^Among people with a positive result for SARS-CoV-2 antibodies

The most prevalent symptoms were cough (*n* = 55, 82.3%) and fever (*n* = 53, 67.7%). The mean number of symptoms was 2.5 (SD 1.0). In most patients (*n* = 52, 76.6%), the symptoms lasted between three and seven days. Differences based on HIV status were not observed for the number (*p* = 0.812) or the duration of symptoms (*p* = 0.204). None of the symptomatic patients required hospitalization. Only one patient reported the persistence of symptoms after the resolution of acute SARS-CoV-2 infection (fatigue and chest/throat pain).

There were no differences in reporting COVID-19 symptoms based on HIV status in both the overall sample (*p* = 0.519) and in the subgroup of patients who tested positive for SARS-CoV-2 antibodies (*p* = 0.715).

### COVID-19: risk factors and testing

Almost all the sample (*n* = 519, 96.1%) reported adopting at least one preventive measure for COVID-19. Those who did not follow preventive measures came mainly from rural areas (*n* = 14, 26%, *p* < 0.001). The type of preventive measures adopted are shown in Table 3. People residing in suburban areas reported most often avoiding crowded areas (42%, *p* < 0.001) and maintaining physical distancing (50%, *p* < 0.001) as preventive measures for COVID-19.

Patients with a previous COVID-19 test were 22 (4.1%); of those, 5 (22.7%) reported being tested positive. Those with a university education or higher underwent a previous COVID-19 test more frequently than the others (primary: *n* = 2, 2.7%, secondary: *n* = 11, 2.8%, university: *n* = 9, 13.8%; *p* = 0.003).

People reporting close contact with a COVID-19 case were 59 (10.9%), and 71 (13.1%) did not remember. Of those with close contact, only 5 (8.5%) underwent a COVID-19 test without differences compared to those without or not remembering (*p* = 0.182).

At least one family member with COVID-19 related-symptoms in the past 8 months was reported by 63 (11.7%) people, and they tested for COVID-19 more frequently (*n* = 7, 11.1%) than those without (*n* = 14, 3.4%, *p* = 0.02). People with a relative with COVID-19 symptoms were more often from urban areas (*n* = 41, 32.8%, *p* < 0001).

## Discussion

This study aimed to explore the prevalence of SARS-CoV-2 antibodies in young people in Beira, Mozambique, which was found to be 46.9% (95%CI 42.6%-51.2%) as of November 2022. The SARS-CoV-2 seroprevalence by HIV status was 35.9% (95%CI 25.3%-47.5%) in YPLHIV and 49.1% (95%CI 44.1%-54.1%) in people without HIV but the difference was not statistically significant.

Although 3 years have passed since the WHO declared the COVID-19 pandemic, data on its impact on Africa are still scarce [15]. The poor accessibility to diagnostic tests has prevented a timely overview of the pandemic’s progression in these countries. Contrary to high-income countries, where the availability of human, material and economic resources made diagnostic tests readily available, in sub-Saharan Africa, this was limited, erratic and in some cases non-existent [16]. In our sample, only 4.1% had a previous COVID-19 test, although some (10.9%) had reported close contact. The number of people who underwent a test having had a relative with COVID-19 symptoms was higher, but still very low, around 11%. Not only the lack of material resources may have contributed to this limited access to SARS-CoV-2 testing. Cultural factors and stigma, often driven by misinformation, also could have played a significant role. In particular, the fear of being shunned by one’s community and evicted from one’s rented home or even from shops and work may have led to testing avoidance and reliance on self-medication [17, 18].

Because of the under-diagnosis challenge, seroprevalence studies are central to tracking the spread of SARS-CoV-2 in the sub-Saharan African population. A systematic review and meta—analysis conducted until May 2022 on seroprevalence studies aligned with the WHO SEROPREV protocol found a global SARS-CoV-2 seroprevalence of 59.2% (95%CI 56.1–62.2). In the WHO African region, seroprevalence increased from 3.5% (95%CI 2.9–4.2) in June 2020 to 86.7% (95%CI 84.6–88.5) in December 2021 due to new and more infectious SARS-CoV-2 variants (i.e., Beta and Omicron) [19]. In our sample, overall seroprevalence was 46.8% (95%CI 42.6–51.2). The latest available data for the city of Beira from 1 to 31 March 2020 reported a seroprevalence of 5.8% [20]. Our findings reflect the Omicron variant’s impact on the region's infection rate. However, a higher prevalence was reported in the third quarter of 2021 in the UN area of Southern Africa, equal to 56.1% (95%CI 44.6–66.9) [5]. The studies in the abovementioned systematic review showed a high heterogeneity of the results. This wide variability can be attributed, on the one hand, to the different study designs, such as the time when it was carried out (i.e., the spread of different variants), the location (urban or rural area), the type of test used, the population involved, and on the other hand to different public health policies adopted, such as non-pharmacological interventions and adherence to prevention measures and vaccination. Moreover, the different seroprevalence reported in our sample may be due to the exclusion of vaccinated people, who were instead considered in the studies mentioned above.

The odd of testing positive was higher among students. Schools are a well-known place of possible contagion. SARS-CoV-2 school-based transmission has been reported in contexts where the level of community infection is high, as is the case in Mozambique after the Beta variant hit the country [21].

Infection probability was also associated with symptoms. The most reported symptoms among SARS-CoV-2 seropositive individuals in our cohort were cough (22.8%), fever (22%), and sore throat (17%), consistent with Omicron predilection for upper respiratory tract infection [22]. The low prevalence of anosmia in our sample also reflects the decreased incidence of this previously more specific sign of SARS-CoV-2 infection during the Omicron era [23]. Symptoms’ duration ranged from 3 to 7 days in most patients; data from a large, observational trial involving self-reporting symptoms among SARS-CoV-2 positive people (16–99 years) reported a median duration of Omicron symptoms of 5 days (IQR 3–9) [23]. Overall, no long-COVID-19 symptoms were experienced by our cohort, except for a single patient who reported fatigue and chest/throat pain but whose duration was not indicated.

Our cohort showed a high prevalence of asymptomatic SARS-CoV-2 infection (71.8%), similar to the high asymptomatic rate reported in other LMICs. A systematic review and meta-analysis showed an asymptomatic prevalence of 71% (IQR 48.4%–80.8%) among SARS-CoV-2 seropositive individuals during the pre-Omicron era in Africa (up to December 2021). However, a possible overestimation of SARS-CoV-2 seroprevalence could have been due to serologic test cross-reaction with other infections [5]. Garrett et al. reported a percentage of 60% of asymptomatic PCR-confirmed Omicron infection in a South African cohort of individuals (mainly YPLHIV) enrolled in a clinical trial [24]. Similarly, our analysis was conducted after the widespread emergence of the Omicron variant from November 2021 in Botswana and South Africa and reflects the milder disease course associated with the Omicron variant compared to the other variants of concern (VOC) [25–28]. In our sample of non-vaccinated young people, only 5 patients (2,1%) reported breathing difficulty, and possible signs of moderate disease, though no one required hospital admission. This high prevalence of asymptomatic carriers accounts, among other factors, for the high transmissibility rate of the Omicron variant, strengthening the need for surveillance and immunization policies, mostly among high-vulnerable populations [29].

No significant differences were found in SARS-CoV-2 seroprevalence between YPLHIV and those without. The relationship between HIV and SARS-CoV-2 co-infection is still uncertain. A study conducted in South Africa after the second wave found a prevalence of 53.2% in YPLHIV with a viral load < 1000 copies/ml and 35.9% in those with a viral load > 1000 copies/ml [30]. The literature is conflicting regarding both the prevalence and severity of COVID-19 in YPLHIV, an increased risk of severe COVID-19 and death, while others found no difference [31]. Our study found no difference concerning symptoms or disease severity between YPLHIV and the others. The mild symptoms and high asymptomatic rate in our HIV-positive cohort may be explained by the characteristics of the sample: young age, low rate of comorbidities and exclusion of those with advanced disease; all these factors were reported to be associated with a better COVID-19 outcome in YPLHIV [30, 32]. Moreover, Omicron infection seems to be related to a high rate of asymptomatic infection, even in HIV-positive people [24].

These differences can result from the combination of several factors in different ways. Co-infection and clinical outcomes are related to individual characteristics, stage of disease, availability and adherence to antiretroviral treatment, personal perception of risk, epidemiological scenario, and use of the correct preventive measures [31]. All these factors may vary geographically and over time, making it difficult to understand their mutual relationship.

Regarding preventive measures, we found a very high knowledge and awareness, over 90%, especially for wearing masks and washing hands, confirming results obtained by a national survey during the first year of the pandemic [33]. Despite the end of the state of emergency, which was declared in Mozambique in March 2022, it is crucial to continue to maintain a high level of adherence to preventive measures, especially in healthcare settings where the risk is increased, having shown high effectiveness in countering the spread of the virus [34].

This study has some limitations. Firstly, no data on HIV viral load and treatment adherence were collected, preventing stratified analyses based on disease status. Furthermore, the number and reason for people excluded based on the inclusion/exclusion criteria was not collected (STROBE checklist in the Additional file). Secondly, the reported COVID-19 symptoms may be affected by recall bias, underestimating them, and SARS-CoV-2 specificity may be difficult to ascertain due to the low execution of the COVID-19 test in this setting. Finally, preventive measures were only investigated in terms of knowledge and awareness, but actual adherence could not be determined.

## Conclusions

The present study contributes to providing data on the seroprevalence of SARS-CoV-2 in Mozambique, which is scarcely documented. Specifically, the SARS-CoV-2 seroprevalence in young people was 46.8% as of November 2022. No differences were observed in the odds of testing positive for SARS-CoV-2 antibodies based on HIV serostatus. A higher risk of contracting the infection was associated with being a student and having COVID-19-related symptoms. This finding supports the role of schools in the spread of infection, even in a setting as little explored as sub-Saharan Africa. Symptoms of COVID-19 were mostly mild with no differences compared to HIV status, although these results may be influenced by the exclusion of YPLHIV with advanced disease. It is important to continue monitoring the spread of the virus and impact of COVID-19 in the general population but particularly in the more fragile groups such as YPLHIV.

### Supplementary Information

**Additional file 1.** Patient questionnaire.

## Data Availability

The datasets generated and/or analysed during the current study are available from the corresponding author on reasonable request.

## References

[1] Unaids. UNAIDS data 2022. Available at: https://www.unaids.org/en/resources/documents/2023/2022_unaids_data. Accessed 11 Apr 2023.

[2] República de Moçambique. Ministério da Saúde, Serviço Nacional de Saúde. Relatório Semestral 2021. Relatório Semestral das Atividades Relacionadas ao HIV/SIDA. Available at: https://www.misau.gov.mz/index.php/relatorios-semestrais. Accessed 13 Apr 2023.

[3] República de Moçambique. Conselho Nacional de Combate ao SIDA. Secretariado Executivo. Sumário do PEN V. 2021–2025. Available at: https://mz.usembassy.gov/wp-content/uploads/sites/182/Apresentacao-do-CNCS-sobre-o-Sumario-do-PEN-V_26-de-Janeiro-de-2021-1.pdf. Accessed 13 Apr 2023.

[4] Ministério da Saúde. Inquérito de Indicadores de Imunização Malária e HIV/SIDA em Moçambique (IMASIDA). Relatório final. 2018. Available at: https://dhsprogram.com/pubs/pdf/AIS12/AIS12.pdf. Accessed 13 Apr 2023.

[5] Lewis HC, Ware H, Whelan M (2022). SARS-CoV-2 infection in Africa: a systematic review and meta-analysis of standardised seroprevalence studies, from January 2020 to December 2021. BMJ Glob Health.

[6] Chisale MRO, Ramazanu S, Mwale SE, Kumwenda P, Chipeta M, Kaminga AC, Nkhata O, Nyambalo B, Chavura E, Mbakaya BC (2022). Seroprevalence of anti-SARS-CoV-2 antibodies in Africa: a systematic review and meta-analysis. Rev Med Virol.

[7] Johns Hopkins University. Center for Systems Science and Engineering. COVID-19 data repository. Avialable at: https://coronavirus.jhu.edu/map.html. Accessed 13 Apr 2023.

[8] República de Moçambique. Ministério da Saúde. Instituto Nacional de Saúde. Relatorio do Inquerito sero-epidemiologico de SARS-CoV-2. Available at: https://www.misau.gov.mz/index.php/covid-19-inquerito-sero-epidemiologicos. Accessed 13 Apr 2023.

[9] WHO Global Clinical Platform for COVID-19. Update on COVID-19/HIV service disruptions. 2021. Available at: https://cdn.who.int/media/docs/default-source/hq-hiv-hepatitis-and-stis-library/2021_hiv_covid_web_1.pdf?sfvrsn=1fc01c6_5. Accessed 13 Apr 2023.

[10] Okegbe T, Williams J, Plourde KF, et al. Impact of COVID-19 on HIV adolescent programming in 16 countries with USAID-supported PEPFAR programs [published online ahead of print, 2023 Mar 29]. J Acquir Immune Defic Syndr. 2023. 10.1097/QAI.0000000000003201. 10.1097/QAI.0000000000003201 PMC10287048 36989134

[11] Bachanas PJ, Chun HM, Mehta N (2022). Protecting the gains: analysis of HIV treatment and service delivery programme data and interventions implemented in 19 African countries during COVID-19. J Int AIDS Soc.

[12] Instituto Nacional De Estatistica. Inidcadores sócio-demográficos. Província de Sofala. 2021. Avialble at: http://www.ine.gov.mz/estatisticas/estatisticas-demograficas-e-indicadores-sociais/perfil-demografico-provincia-de-sofala.pdf/view. Accessed 13 Apr 2023.

[13] Abbott. PanbioTM COVID-19 IgG/IgM Rapid Test Device (fingerstick whole blood/venous whole blood/serum/plasma) instructions for use. 2020. REF ICO-T402 / ICO-T40203. Available at: https://www.globalpointofcare.abbott/en/product-details/panbio-covid-19-igg-igm-antibody-test.html. Accessed 13 Apr 2023.

[14] Harris PA, Taylor R, Minor BL, Elliott V, Fernandez M, O’Neal L, McLeod L, Delacqua G, Delacqua F, Kirby J, Duda SN, REDCap Consortium (2019). The REDCap consortium: building an international community of software partners. J Biomed Inform.

[15] World Health Organization. Regional Office for Europe. A timeline of WHO’s COVID-19 response in the WHO European region: a living document (version 3.0, from 31 December 2019 to 31 December 2021). World Health Organization. Regional Office for Europe; 2022. Available at: https://apps.who.int/iris/handle/10665/351782. Accessed 13 Apr 2023.

[16] Oleribe OO, Suliman AAA, Taylor-Robinson SD, Corrah T (2021). Possible reasons why Sub-Saharan Africa experienced a less severe COVID-19 pandemic in 2020. J Multidiscip Healthc.

[17] Peprah P, Gyasi RM (2021). Stigma and COVID-19 crisis: a wake-up call. Int J Health Plann Manage.

[18] Jarolimova J, Yan J, Govere S (2021). Medical mistrust and stigma associated with COVID-19 among people living with HIV in South Africa. AIDS Behav.

[19] Bergeri I, Whelan MG, Ware H (2022). Global SARS-CoV-2 seroprevalence from January 2020 to April 2022: a systematic review and meta-analysis of standardized population-based studies. PLoS Med.

[20] Observatorio National de Saude (ONS). COVID-19 em Moçambique Relatório do 1° Ano 2020–2021. Avialable at: https://www.misau.gov.mz/index.php/covid-19-em-mocambique-relatorios. Accessed 13 Apr 2023.

[21] Hargreaves JR, Langan SM, Oswald WE (2022). Epidemiology of SARS-CoV-2 infection among staff and students in a cohort of English primary and secondary schools during 2020–2021. Lancet Reg Health Eur.

[22] Nori W, Ghani Zghair MA (2022). Omicron targets upper airways in pediatrics, elderly and unvaccinated population. World J Clin Cases.

[23] Menni C, Valdes AM, Polidori L (2022). Symptom prevalence, duration, and risk of hospital admission in individuals infected with SARS-CoV-2 during periods of omicron and delta variant dominance: a prospective observational study from the ZOE COVID Study. Lancet.

[24] Garrett N, Tapley A, Andriesen J, Seocharan I, Fisher L, Bunts L, Espy N, Wallis C, Randhawa A, Ketter N (2022). High asymptomatic carriage with the Omicron variant in South Africa. Clin Infect Dis.

[25] Tegally H, Wilkinson E, Giovanetti M (2021). Detection of a SARS-CoV-2 variant of concern in South Africa. Nature.

[26] Iuliano AD, Brunkard JM, Boehmer TK, Peterson E, Adjei S, Binder AM, Cobb S, Graff P, Hidalgo P, Panaggio MJ, Rainey JJ, Rao P, Soetebier K, Wacaster S, Ai C, Gupta V, Molinari NM, Ritchey MD (2022). Trends in disease severity and health care utilization during the early Omicron variant period compared with previous SARS-CoV-2 high transmission periods - United States, December 2020-January 2022. MMWR Morb Mortal Wkly Rep.

[27] Esper FP, Adhikari TM, Tu ZJ, Cheng YW, El-Haddad K, Farkas DH, Bosler D, Rhoads D, Procop GW, Ko JS, Jehi L, Li J, Rubin BPSO (2023). Alpha to Omicron: disease severity and clinical outcomes of major SARS-CoV-2 variants. J Infect Dis.

[28] Jassat W (2022). Clinical severity of COVID-19 in patients admitted to hospital during the omicron wave in South Africa: a retrospective observational study. Lancet Glob Health.

[29] COVID-19 Treatment Guidelines Panel. Coronavirus disease 2019 (COVID-19) treatment guidelines. National Institutes of Health. Available at https://www.covid19treatmentguidelines.nih.gov/. Accessed 20 Mar 2023. 34003615

[30] Wolter N, Tempia S, von Gottberg A (2022). Seroprevalence of severe acute respiratory syndrome coronavirus 2 after the second wave in South Africa in human immunodeficiency virus-infected and uninfected persons: a cross-sectional household survey. Clin Infect Dis.

[31] Oyelade T, Alqahtani JS, Hjazi AM, Li A, Kamila A, Raya RP (2022). Global and regional prevalence and outcomes of COVID-19 in people living with HIV: a systematic review and meta-analysis. Trop Med Infect Dis.

[32] Dandachi D, Geiger G, Montgomery MW (2021). Characteristics, comorbidities, and outcomes in a multicenter registry of patients with human immunodeficiency virus and coronavirus disease 2019. Clin Infect Dis.

[33] Júnior A, Dula J, Mahumane S (2021). Adherence to COVID-19 preventive measures in Mozambique: two consecutive online surveys. Int J Environ Res Public Health.

[34] Talic S, Shah S, Wild H (2021). Effectiveness of public health measures in reducing the incidence of covid-19, SARS-CoV-2 transmission, and covid-19 mortality: systematic review and meta-analysis [published correction appears in BMJ. 2021 Dec 3;375:n2997]. BMJ.

