# Supplementary material for: SARS-CoV-2 seroprevalence and associated factors, based on HIV serostatus, in young people in Sofala province, Mozambique
Source: BMC Infect Dis. 2023 Nov 17;23:809. doi: 10.1186/s12879-023-08808-6 (PMC10656907; doi:10.1186/s12879-023-08808-6)
Supplement: Supplementary file 1 — Additional file 1. Patient questionnaire. [file 12879_2023_8808_MOESM1_ESM.docx]

**ADDITIONAL FILE**

**PATIENT QUESTIONNAIRE**

**SECTION I: Inclusion criteria**

A1. The user refuses to participate in the study?

a. Yes (Not eligible for study)

b. No

*The clinical officer (physician, nurse, health technician) will proceed with the administration of the questionnaire ONLY IF the answer to ALL of the following questions is NO (this does not apply to HIV positivity questions)*

A2. Is the user under 18 years of age OR over 24 years of age?

a. Yes (Not eligible for study

b. No

A3. Is the user resident outside the city of Beira and the district of Nhamatanda?

a. Yes (Not eligible for study)

b. No

A4. Does the patient have symptoms of COVID-19 today?

a. Yes (Not eligible for study)

b. No

A5. Has the patient been vaccinated for COVID-19?

a. Yes (Not eligible for study)

b. No

A6. Is the user pregnant?

a. Yes (Not eligible for study)

b. No

*If the user is HIV positive in follow-up, tick "HIV positive user" below. If the user was tested for HIV less than 6 months ago, tick "HIV negative user" below. If not (the user tested for HIV more than 6 months ago or never tested), offer testing today before ticking "HIV negative user" below.*

A7. HIV positive user

A7.1. Does the patient have advanced HIV/AIDS with active diseases (WHO clinical stage III-IV or

CD4 < 200/< 15%)?

a. Yes (Not eligible for study)

b. No

A7.2 Has the user been on ART for less than 6 months?

a. Yes (Not eligible for study)

b. No

A8. HIV negative user

A8.1 Date of last negative HIV test (valid if it was taken within the last 6 months)________

**SECTION II: Sociodemographic Characteristics**

(Please mark with “x” the participant's answers)

1. Name of the Health Unit

1. Munhava

2. Macurungo

3. Ponta Gea

4. Inhamizua

5. Nhaconjo

6. Hospital Central Beira

7. Mascarenas

8. Chingussura

9. HR Nhamatanda

2. ID: ___________________

3. Sex

a. Male

b. Female

c. Other, specify ______________

4. Age (enter an integer): ________________

5. Level of education (if not graduated or postgraduated, indicate in number of the class that the user is attending or has already attended)

1. Primary level: ___

2. Mid-level: ___

3. Upper level: ___

4. Postgraduate: ___

6. Is the participant currently studying?

1. Yes, exclusively

2. Yes, study and work

3. No

7. What is the participant's profession?

1. Unemployed

2. Informal employment

3. Wage worker

4. State employee

5. Private employee

6. Farmer (as main source of income)

7. Other, please specify

8. What is your marital status?

1. Single

2. De facto union

3. Married

4. Widow/widower

5. Other, specify

9. Residence:

1. Macuti 14. Alto from Manga-Nhaconjo

2. Palm trees 15. Chingussura

3. Gea Point 16. Vila Massane

4. Chaimite 17. Inhamizua

5. Pioneers 18. Slaughterhouse

6. Esturro 19. Mungassa

7. Matacuane 20. Ndunda

8. Macurungo 21. Manga-Mascarenha

9. Munhava Central 22. Muave

10. Mananga 23. Nhangau

11. Vaz 24. Nhangoma

12. Maraza 25. Tchonja

13. Chota 26. Dstrito of Nhamatanda

10. Does the participant have children?

1. Yes, how many (specify _____)

2. No

11. Household (specify the number of persons) _______

12. What is participant religion?

1. Catholic

2. Islamic

3. Anglican

4. Zione/Zion

5. Protestant (Evangelical/Pentecostal)

6. None

7. Don't know

8. Other, specify

**SECTION III: Associated factors**

13. Had the participant been using COVID-19 preventive measures for the past 8 months?

1. Yes

2. No

14. If so, which of these measures?

1. Mask use

2. Physical distancing

3. Avoid crowds

4. Hand washing

5. Open the windows

6. Other, please specify

15. How many people live with the participant? _______

16. How many rooms does the participant have in his/her house? ______

17. Has the participant had close contact with a suspected or confirmed cases of COVID-19 in the last 8 months?

a. Yes

b. Not

c. I don't remember/know

18. Has the participant had any family member with symptoms of COVID-19 in the last 8 months?

a. Yes

b. No

c. I don't remember/know

19. What are the comorbidities of the participant (tick one or more answers)

1. Active tuberculosis

2. Hypertension

3. Diabetes

4. Cancer

5. Asthma/COPD

6. Obesity/overweight

7. None

8. Other, please specify: ____________

**SECTION IV: Clinical Manifestations and Outcome**

20. Has the participant been tested for COVID-19 in the last 8 months?

1. Yes

2. No

22. What was the result of the test?

1. Positive

2. Negative

23. Has the participant had suspected symptoms of COVID-19 in the last 8 months?

1. Yes

2. No

24. Symptoms:

1. Fever

2. Cough

3. Sore throat

4. Anosmia/ageusia (loss of smell and/or taste)

5. Difficulty breathing

25. If so, how long did the symptoms last?

1. Less than 3 days

2. Between 3 and 7 days

3. More than 7 days

26. Was the participant admitted to the hospital?

1. Yes

2. No

27. If so, how long the hospitalization lasted?

1. Less than 3 days

2. Between 3 and 7 days

3. More than 7 days

28. Why were the participants admitted to the hospital?

1. For the disease caused by COVID-19

2. For another disease (specify)

1. Malaria

2. Respiratory disease/pneumonia

3. Gastroenteritis/diarrhoea

4. Urinary infection

5. Other (please specify):_______________

29. When did the participant test negative after the onset of symptoms?

a. After 10 days

b. After 15 days

c. > 15 days

d. I don't remember

30. After acute COVID infection, did these symptoms remain? (More than one answer is possible)

1. Difficulty breathing

2. Cough

3. Fever

4. Changes in smell and taste

5. Tiredness

6. Chest/throat pain

7. Headache

8. Abdominal pain/diarrhoea

9. Muscle aches

10. Anxiety/depression

11. Insomnia

**SECTION V: SARS-COV-2 test result**

1. Positive (IgM only)

2. Positive (IgG only)

3. Positive (IgM + IgG)

4. Negative

5. Invalid

Did you explain the result to the participant?

a. Yes

b. No

**STROBE Statement—Checklist of items that should be included in reports of cross-sectional** **studies**.

|  | Item No | Recommendation | Page  No. |
| --- | --- | --- | --- |
| **Title and abstract** | 1 | (*a*) Indicate the study’s design with a commonly used term in the title or the abstract | 2 |
|  |  | (*b*) Provide in the abstract an informative and balanced summary of what was done and what was found | 2 |
| Introduction | | |  |
| Background/rationale | 2 | Explain the scientific background and rationale for the investigation being reported | 3 |
| Objectives | 3 | State specific objectives, including any prespecified hypotheses | 3 |
| Methods | | |  |
| Study design | 4 | Present key elements of study design early in the paper | 4 |
| Setting | 5 | Describe the setting, locations, and relevant dates, including periods of recruitment, exposure, follow-up, and data collection | 4 |
| Participants | 6 | (*a*) Give the eligibility criteria, and the sources and methods of selection of participants | 4 |
| Variables | 7 | Clearly define all outcomes, exposures, predictors, potential confounders, and effect modifiers. Give diagnostic criteria, if applicable | 5 |
| Data sources/ measurement | 8* | For each variable of interest, give sources of data and details of methods of assessment (measurement). Describe comparability of assessment methods if there is more than one group | 5 |
| Bias | 9 | Describe any efforts to address potential sources of bias | 4 |
| Study size | 10 | Explain how the study size was arrived at | 4 |
| Quantitative variables | 11 | Explain how quantitative variables were handled in the analyses. If applicable, describe which groupings were chosen and why | 5 |
| Statistical methods | 12 | (*a*) Describe all statistical methods, including those used to control for confounding | 5 |
|  |  | (*b*) Describe any methods used to examine subgroups and interactions | 5 |
|  |  | (*c*) Explain how missing data were addressed | 5 |
|  |  | (*d*) If applicable, describe analytical methods taking account of sampling strategy |  |
|  |  | (*e*) Describe any sensitivity analyses | NA |
| Results | | |  |
| Participants | 13* | (a) Report numbers of individuals at each stage of study—eg numbers potentially eligible, examined for eligibility, confirmed eligible, included in the study, completing follow-up, and analysed | 6 |
|  |  | (b) Give reasons for non-participation at each stage | NA |
|  |  | (c) Consider use of a flow diagram | NA |
| Descriptive data | 14* | (a) Give characteristics of study participants (eg demographic, clinical, social) and information on exposures and potential confounders | 6 |
|  |  | (b) Indicate number of participants with missing data for each variable of interest | 6 |
| Outcome data | 15* | Report numbers of outcome events or summary measures | 6 |
| Main results | 16 | (*a*) Give unadjusted estimates and, if applicable, confounder-adjusted estimates and their precision (eg, 95% confidence interval). Make clear which confounders were adjusted for and why they were included | 14 |
|  |  | (*b*) Report category boundaries when continuous variables were categorized | NA |
|  |  | (*c*) If relevant, consider translating estimates of relative risk into absolute risk for a meaningful time period. | NA |
| Other analyses | 17 | Report other analyses done—eg analyses of subgroups and interactions, and sensitivity analyses | 6 |
| Discussion | | |  |
| Key results | 18 | Summarise key results with reference to study objectives | 7 |
| Limitations | 19 | Discuss limitations of the study, taking into account sources of potential bias or imprecision. Discuss both direction and magnitude of any potential bias | 8 |
| Interpretation | 20 | Give a cautious overall interpretation of results considering objectives, limitations, multiplicity of analyses, results from similar studies, and other relevant evidence | 7,8 |
| Generalisability | 21 | Discuss the generalisability (external validity) of the study results | 7,8 |
| Other information | | |  |
| Funding | 22 | Give the source of funding and the role of the funders for the present study and, if applicable, for the original study on which the present article is based | 9 |

*Give information separately for exposed and unexposed groups.

**Note:** An Explanation and Elaboration article discusses each checklist item and gives methodological background and published examples of transparent reporting. The STROBE checklist is best used in conjunction with this article (freely available on the Web sites of PLoS Medicine at http://www.plosmedicine.org/, Annals of Internal Medicine at http://www.annals.org/, and Epidemiology at http://www.epidem.com/). Information on the STROBE Initiative is available at www.strobe-statement.org.
